# Subject-Specific Pressure Normalization of Local Pulse Wave Velocity: Separating Intrinsic From Acute Load-Dependent Stiffening in Hypertensive Patients

**DOI:** 10.3389/fphys.2021.783457

**Published:** 2022-02-15

**Authors:** Alessandro Giudici, Carlo Palombo, Michaela Kozakova, Carmela Morizzo, J. Kennedy Cruickshank, Ashraf W. Khir

**Affiliations:** ^1^Brunel Institute for Bioengineering, Brunel University London, Uxbridge, United Kingdom; ^2^Department of Surgical, Medical, Molecular Pathology and Critical Area Medicine, University of Pisa, Pisa, Italy; ^3^Department of Clinical and Experimental Medicine, University of Pisa, Pisa, Italy; ^4^School of Life-Course/Nutritional Sciences, King’s College, St. Thomas’ and Guy’s Hospitals, London, United Kingdom

**Keywords:** pressure-independent PWV, hypertension, arterial stiffness, common carotid arteries, blood pressure

## Abstract

Pulse wave velocity (PWV) is a powerful predictor of cardiovascular events. However, its intrinsic blood pressure (BP)-dependency complicates distinguishing between acute and chronic effects of increased BP on arterial stiffness. Based on the assumption that arteries exhibit a nearly exponential pressure-area (*P*-*A*) relationship, this study proposes a method to assess intersubject differences in local PWV independently from BP. The method was then used to analyze differences in local carotid PWV (cPWV) between hypertensive and healthy normotensive people before and after BP-normalization. Pressure (*P*) and diameter (*D*) waveforms were simultaneously acquired *via* tonometer at the left and ultrasound scanning at right common carotid artery (CCA), respectively, in 22 patients with Grade 1 or 2 hypertension and 22 age- and sex-matched controls. cPWV was determined using the *D*^2^*P*-loop method. Then, the exponential modeling of the *P*-area (*A* = π*D*^2^/4) relationships allowed defining a mathematical formulation to compute subject-specific changes in cPWV associated with BP changes, thus enabling the normalization of cPWV against intersubject differences in BP at the time of measurement. Carotid systolic BP (SBP) and diastolic BP (DBP) were, on average, 17.7 (*p* < 0.001) and 8.9 mmHg (*p* < 0.01) higher in hypertensives than controls, respectively. cPWV was 5.56 ± 0.86 m/s in controls and 6.24 ± 1.22 m/s in hypertensives. BP alone accounted for 68% of the cPWV difference between the two groups: 5.80 ± 0.84 vs. 6.03 ± 1.07 m/s after BP-normalization (*p* = 0.47). The mechanistic normalization of cPWV was in agreement with that estimated by analysis of covariance (ANCOVA). In conclusion, the proposed method, which could be easily implemented in the clinical setting, allows to assess the intersubject differences in PWV independently of BP. Our results suggested that mild hypertension in middle-aged subjects without target organ damage does not significantly alter the stiffness of the CCA wall independently of acute differences in BP. The results warrant further clinical investigations to establish the potential clinical utility of the method.

## Introduction

Arterial stiffness as pulse wave velocity (PWV) is a powerful predictor of mortality and cardiovascular events in hypertensive patients, above and beyond traditional risk factors (Boutouyrie et al., 2002; Laurent et al., 2003; Cardoso et al., 2019). The arterial wall, however, presents a complex microstructure where different wall constituents, mainly collagen, elastin, and smooth muscle, play different but equally important roles in arterial function (Boutouyrie et al., 1998; Cruickshank et al., 2002; Krasny et al., 2017; Giudici et al., 2021e). The heterogeneous microstructure of the arterial wall makes its behavior highly nonlinear (Giudici et al., 2021b) so that arterial stiffness and, consequently, PWV are intrinsically blood pressure (BP)-dependent (Spronck et al., 2015b). This fact complicates distinguishing between chronic (i.e., actual BP-induced wall remodeling) and acute effects (i.e., transitional shift to a different working point in the nonlinear behavior of the arterial wall) of increased BP on arterial structure and mechanics. Most clinical studies address the issue of the BP-dependency of PWV *via* statistical methods, using BP as a confounding factor for PWV (Desamericq et al., 2015; Diaz et al., 2018; Valbusa et al., 2019). However, statistical methods suffer some limitations: (1) they lack subject specificity and, hence, are less likely to be used in clinical practice, (2) they may fail to discriminate between acute and chronic effects of increased BP on the wall stiffness (Spronck, 2021), and (3) the choice of the normalizing pressure, i.e., the subject-specific pressure level to be used as the confounder in multivariate analysis, is not trivial and largely affects the size of the correction itself (Giudici et al., 2021a). While, in clinical investigations, statistical adjustments for systolic BP (SBP) (Valbusa et al., 2019), mean BP (MBP) (Desamericq et al., 2015), and pulse pressure (PP) (Brandts et al., 2012) are often made, most regional PWV metrics (e.g., carotid-femoral and brachial-ankle PWV), use the foot of arterial waves as the fiducial point, suggesting that diastolic BP (DBP) likely represent a more appropriate choice for their pressure-normalization (Spronck et al., 2017b). As intergroup differences in SBP are typically larger than those in DBP, widely used SBP- and MBP-statistical adjustments likely lead to overcorrections of PWV and potentially limit our understanding of pressure-induced chronic vascular damage (Spronck et al., 2017b).

In the past two decades, researchers have attempted to address the BP-dependency of PWV using mechanistic approaches that rely chiefly on the assumption that, in the physiological pressure range, the pressure (*P*)-area (*A*)/diameter (*D*) relationship of arteries resembles an exponential function (Gavish and Izzo, 2016). If the fitting exponential function is opportunely formulated, its constant can be used as a BP-normalized index of arterial stiffness (Spronck et al., 2017a; Giudici et al., 2021a). In 2006, Shirai et al. introduced the cardio-ankle vascular index (CAVI) that uses the heart-to-ankle PWV (haPWV), a regional PWV metric quantifying the average properties of the entire heart-to-ankle arterial pathway, to estimate Kawasaki’s stiffness index β, defining the exponential relationship between *P* and *D*. A decade later, however, Spronck et al. (2017a) raised concerns related to the actual (in)dependency of CAVI from BP at the time of measurement, due to the inherent dependency of β from the subject-specific DBP. They proposed a revised metric CAVI_0_ that relies on Hayashi’s stiffness index β_0_, which, unlike β, is defined with respect to a standardized reference pressure (*P*_ref_). Furthermore, CAVI and CAVI_0_ differ also in the normalizing pressure used to estimate β from haPWV, raising once more the question of which is the most appropriate pressure level for the normalization of PWV metrics (Giudici et al., 2021a).

Similar methods have been devised to correct local PWV estimates, which, unlike regional PWVs, provide information on the stiffness of a specific location of the arterial tree. Spronck et al. (2015a) used the exponential *P-A* relationship proposed by Meinders and Hoeks (2004) to effectively predict the changes in local carotid PWV (cPWV), estimated *via* a linearized Bramwell-Hill equation (Bramwell et al., 1923), associated with the BP lowering achieved with 3-months of antihypertensive treatment in hypertensive patients. A considerably more complex approach was proposed by Ma et al. (2018) who used Fung’s exponential hyperelastic strain energy function to define the relationship between BP and local PWV. While undoubtedly elegant, this approach is unlikely to be used in clinical practice due to the difficulty in estimating subject-specific parameters for the mathematical model describing the artery behavior.

Using a similar mechanistic approach based on the exponential modeling of the *P*-*A* relationship of arteries (Spronck et al., 2015b,2017a), our current study analyzed differences in local cPWV between healthy controls and patients with hypertension, aiming to distinguish between acute and chronic effects of high BP on carotid function. We also aimed to compare mechanistic and statistical correction of cPWV, analyzing the impact of the choice of the normalizing pressure on the size of the correction and on inter-group differences.

## Materials and Methods

### Theoretical Background

Tube laws mathematically define the relationship between *P* and *A* in flexible tubes. The pressure-normalization method adopted in this study is based on the tube law proposed by Meinders and Hoeks (2004):


(1) $$\begin{array}{c} P\,\left(D\right)=P_{\text{ref}}\,{\text{e}}^{\gamma_{0}\,\left(\frac{D^{2}}{D_{\text{ref}}^{2}}-1\right)}, \end{array}$$

where *D* is the luminal diameter (*A* is assumed to be circular, *A* = π*D*^2^/4), *P*_ref_ is a reference pressure, *D*_ref_ is the diameter at *P*_ref_, and γ_0_ is an index of arterial stiffness defining the exponential relationship between *P* and *D*^2^. Note that identical *P*-*D*^2^ relationships can be obtained with opportunely different combinations of *P*_ref_, *D*_ref_, and γ_0_ (Giudici et al., 2021a). Eq. 1 represents a generalized form of the tube law proposed by Meinders and Hoeks who chose *P*_ref_ = DBP. While *P*_ref_ does not have any physiological meaning and its choice is arbitrary, fixing *P*_ref_ to a constant value makes γ_0_ a pressure-normalized index of arterial stiffness. Following previous studies (Giudici et al., 2021a; van der Bruggen et al., 2021), we chose *P*_ref_ = 100 mmHg. This value represents the most commonly established value for mean pressure of healthy adults and allows direct comparison with previous studies.

The Bramwell-Hill equation (Bramwell et al., 1923), defining the relationship between arterial distensibility and local PWV, suggests that PWV at a given pressure level *P*_*c*_ can be expressed as a function of the slope of the tangent to the *P*-*D*^2^ relationship at *P*_*c*_.


(2) $$\begin{array}{c} \text{PWV}\,\left(P_{c}\right)=\sqrt{{\frac{D^{2}}{\rho }\,\frac{\text{d}\,P}{\text{d}\,D^{2}}|}_{P_{c}}}, \end{array}$$

where ρ is the blood density, here assumed as 1,060 kg/m^3^. Using Eq. 1 to determine the derivative term in Eq. 2 and rearranging using Eq. 1 leads to the following relationship between PWV and γ_0_, as previously demonstrated (Giudici et al., 2021d) (refer to Eqs A1–A7 in Appendix for the detailed calculations):


(3) $$\begin{array}{c} \text{PWV}\,{\left(P_{c}\right)}^{2}=\frac{P_{c}\,\gamma_{0}}{\rho }+\frac{P_{c}}{\rho }\,\ln\left(\frac{P_{c}}{P_{\text{ref}}}\right). \end{array}$$

Eq. 3 indicates that PWV at any *P*_*c*_ can be directly estimated using γ_0_ and *P*_ref_.

Let us define a target pressure *P*_T_ as the pressure level to which normalization is required. Given that PWV was measured at the pressure *P*_*c*_ [i.e., PWV(*P*_*c*_)], the pressure change used to normalize PWV(*P*_*c*_) is determined as the difference between the two pressure levels: *P*_T_–*P*_*c*_. Following from Eq. 3, PWV at *P*_T_ can be determined as follows:


(4) $$\begin{array}{c} \mathrm{PWV}\,{\left(P_{\text{T}}\right)}^{2}=\frac{P_{\text{T}}\,\gamma_{0}}{\rho }+\frac{P_{\text{T}}}{\rho }\,\ln\left(\frac{P_{\text{T}}}{P_{\text{ref}}}\right). \end{array}$$

By combining Eq. 3 and 4, we obtain as follows:


(5) $$\begin{array}{c} \text{PWV}\,\left(P_{\text{T}}\right)=\sqrt{\text{PWV}\,{\left(P_{c}\right)}^{2}\,\frac{P_{\text{T}}}{P_{c}}+\frac{P_{\text{T}}}{\rho }\,\ln\left(\frac{P_{\text{T}}}{P_{c}}\right)}. \end{array}$$

Eq. 5 allows us to convert PWV at pressure *P*_*c*_ to the desired target pressure *P*_T_. We noted that *P*_*c*_ is generally unknown and depends on the choice of the method used to estimate PWV. However, if γ_0_ is known, *P*_*c*_ for any PWV estimation method can be numerically estimated by solving Eq. 3.

### Study Population and Data Acquisition

The study sample came from individuals undergoing standard outpatient cardiovascular risk assessment at the Pisa University Hospital (Pisa, Italy), omitting anyone with carotid atherosclerotic plaque, diabetes, and history of major cardiovascular events, atrial fibrillation, malignancy, or chronic inflammatory disease. Hypertension was defined as brachial SBP (SBP_b_) > 140 mmHg and/or DBP > 90 mmHg or based on active antihypertensive treatment. The final study population included *n* = 22 healthy normotensive controls and *n* = 22 patients with mild-to-moderate (or Grade 1–2) hypertension (Williams et al., 2018), of which 41% (*n* = 9) were treated (treatment duration < 1 year). The protocol of the study followed the principles of the Declaration of Helsinki and was approved by the institutional ethics committee “Comitato Etico di Area Vasta Nord Ovest” (reference number: 3146/2010). All subjects gave their informed consent to participate.

**FIGURE 1:**
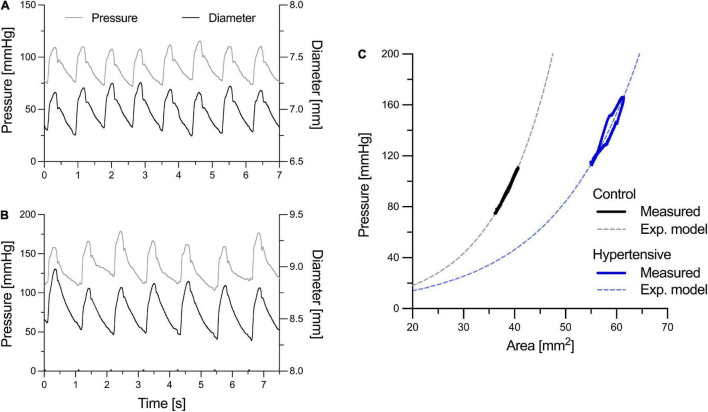
Examples of left common carotid artery (CCA) pressure and right CCA diameter distension waveforms of a representative normotensive (control) person **(A)** and hypertensive patient **(B)**. Pressure and diameter waveforms were aligned using the two major peaks of their second derivative, representing the foot of the wave and the dicrotic notch, as fiducial points. In **(C)**, representative pressure-area (*P*-*A*) relationships were obtained by ensemble averaging pressure and diameter heartbeats [the luminal area (*A*) was calculated from the diameter *(D)* assuming a circular luminal area: *A* = π*D*^2^/4]. **(C)** Illustrates how the exponential relationship in Eq. 1 was fitted on the measured subject-specific *P*-*A* relationships to estimate the stiffness index γ_0_ (notably, in reality, this was performed on a beat-to-beat basis rather than on the ensemble-averaged curves).

Both SBP_b_ and DBP were measured using a digital Omron device (model 705cp, Kyoto, Japan) after subjects had rested for at least 15 min in the supine position. Then, the pressure waveform of the left common carotid artery (CCA) and the diameter waveform of the right CCA were simultaneously acquired by tonometry (PulsePen, DiaTecne, Milan, Italy; sampling frequency = 1 kHz) and ultrasound scanning (Aloka Prosound 10, Hitachi Ltd., Japan), respectively. A similar coupling of waveforms acquired at the contralateral CCAs has been performed previously to achieve correspondence in heartbeats between the pressure and diameter signals, under the assumption that hemodynamic features are similar in the two CCAs due to their similar geometrical characteristics and downstream branching (Giannattasio et al., 2008). The ultrasound machine was equipped with a 10.0 MHz linear array probe with radiofrequency data output at the frequency of 1 kHz. In the longitudinal right CCA view, a single scan line was aligned perpendicularly to the vessel walls, approximately 1.5 cm proximal to the carotid bulb, as reported previously (Uejima et al., 2019). The cursors were then placed by the experienced operator at the anterior and posterior carotid walls to enable wall tracking. PulsePen recordings were calibrated assuming constant DBP and MBP along the arterial tree. Brachial MBP was estimated from SBP_b_ and DBP assuming a form factor of 0.43: MBP = DBP+0.43 (SBP_b_–DBP) (Segers et al., 2009). Both acquisitions lasted for approximately 10 s, yielding 7–10 overlapping pressure and diameter cardiac cycles to be used in the analysis. The two main peaks of the second derivatives (i.e., acceleration) of the pressure and diameter signals (identifying the foot of the wave and dicrotic notch, respectively) were used as fiducial points for the alignment of the two waveforms, as previously described (Giudici et al., 2021c). Figures 1A,B provide examples of aligned left CCA pressure and right CCA diameter waveforms in a representative normotensive and hypertensive person, respectively.

### Data Analysis

cPWV was estimated using the *D*^2^*P*-loop method (Alastruey, 2011), a linearization of the Bramwell-Hill equation (Eq. 2) over the late diastolic part of the *P*-*D*^2^ relationship (i.e., the diastolic decay spanning from the pressure at the dicrotic notch, *P*_notch_, to DBP), as described previously (Giudici et al., 2021c).


(6) $$\begin{array}{c} \text{cPWV}=\sqrt{\frac{D_{\text{d}}^{2}}{\rho }\,\frac{\text{d}\,P}{\text{d}\,D^{2}}} \end{array}$$

where *D*_d_ is the diameter at DBP, and the derivative term d*P*/d*D*^2^ indicates the slope of the linear regression of the late diastolic part of the *P*-*D*^2^ relationship.

As detailed in the theoretical background section, the accurate BP-normalization of cPWV requires knowledge of the pressure, *P*_*c*_, that drives the BP-dependency of cPWV estimated with the *D*^2^*P*-loop method. As indicated above, the *D*^2^*P*-loop method estimates cPWV over the pressure range between *P*_notch_ and DBP. Therefore, the representative *P*_*c*_ is expected to fall within these two pressure levels. To estimate the most suitable *P*_*c*_ for the *D*^2^*P*-loop method, the beat-to-beat relationships between left CCA *P* and right CCA *D*^2^ were fitted using Eq. 1, as shown in Figure 1C, to estimate γ_0_. For each heartbeat, this step entailed determining the combination of γ_0_ and *D*_ref_ that minimizes the difference between the measured *P* waveform and that estimated using Eq. 1, given the *D* waveform as input. Then, the most suitable *P*_*c*_ was numerically determined by solving Eq. 3, given γ_0_ and cPWV. Notably, for each patient, the used γ_0_ and cPWV were the mean values of the 7–10 simultaneously recorded heartbeats. Finally, cPWV was pressure-normalized using Eq. 5.

### Statistical Analysis

Statistical analysis was performed using SPSS 23 (SPSS, IBM Corp., Chicago, IL, United States). Comparison of variables between the two groups was first performed using Student’s *t*-test and then adjusting for potential confounders using analysis of covariance (ANCOVA). The BP-normalization of cPWV was performed using both mechanistic (Eq. 5) and statistical (ANCOVA) methods and considering four different pressure levels as normalizing pressure: (1) *P*_*c*_, determined numerically by solving Eq. 3, (2) DBP, (3) MBP, and (4) SBP. The latter three pressure levels were chosen because often used as normalizing pressures in clinical studies. For each pressure level, *P*_T_ in Eq. 5 was set to the average *P*_*c*_, DBP, MBP, or SBP across groups. Data are generally presented as average ± standard deviation (SD). ANCOVA estimates are presented as average (95% CI). The *p*-value < 0.05 was considered statistically significant.

## Results

### Group Characteristics

Group characteristics are presented in Table 1. Hypertensive and control groups were matched in age and gender, but not heart rate (HR) that was on average 6 bpm higher in hypertensives (*p* = 0.021). Carotid SBP was, on average 17.7 mmHg higher in hypertensives than controls (*p* < 0.001), while the difference in DBP was approximately half (*p* < 0.01). As a result, PP was also 8.7 mmHg higher in hypertensives than controls. Average CCA diameters at SBP and DBP were slightly higher in hypertensives than controls, but differences were not significant and were reduced further after appropriate SBP and DBP adjustments [7.62 (7.23–8.02) vs. 7.76 (7.37–8.16) mm and 7.15 (6.77–7.53) vs. 7.33 (6.95–7.71) mm]. Conversely, carotid IMT and IMT/*D*_d_ were higher in hypertensives than controls (*p* = 0.005 and *p* = 0.058, respectively).

**TABLE 1:** Characteristics and carotid artery dimensions of control and hypertension groups.

	Controls	Hypertensives	
Age [years]	55 ± 6	56 ± 9	*p* = 0.77
Male : Females	13 : 9	13 : 9	*p* = 0.77
HR [bpm]	60 ± 7	66 ± 9	*p* = 0.021
SBP_b_ [mmHg]	116.6 ± 12.2	133.7 ± 17.9	*p* < 0.001
SBP [mmHg]	113.9 ± 11.4	131.6 ± 17.9	*p* < 0.001
DBP [mmHg]	74.9 ± 8.7	83.8 ± 9.8	*p* = 0.003
MBP [mmHg]	92.7 ± 9.5	105.1 ± 12.2	*p* < 0.001
PP [mmHg]	39.0 ± 7.4	47.7 ± 12.6	*p* = 0.009
*P*_notch_ [mmHg]	100.0 ± 9.9	114.6 ± 14.6	*p* < 0.001
*D*_s_ [mm]	7.54 ± 0.84	7.85 ± 0.82	*p* = 0.23
*D*_d_ [mm]	7.08 ± 0.83	7.40 ± 0.81	*p* = 0.21
IMT [μm]	711 ± 145	819 ± 138	*p* = 0.005
IMT/*D*_d_ [–]	0.101 ± 0.017	0.111 ± 0.015	*p* = 0.058

*The p-values are the results of the independent Student’s t-test.*

*D_d_, diastolic diameter; D_s_, systolic diameter; DBP, diastolic blood pressure; HR, heart rate; IMT, intima-media thickness; MBP, mean blood pressure; P_notch_, pressure at the dicrotic notch; PP, pulse pressure; SBP, carotid systolic blood pressure; SBP_b_, brachial systolic blood pressure. Data are presented as mean ± standard deviation (SD).*

**FIGURE 2:**
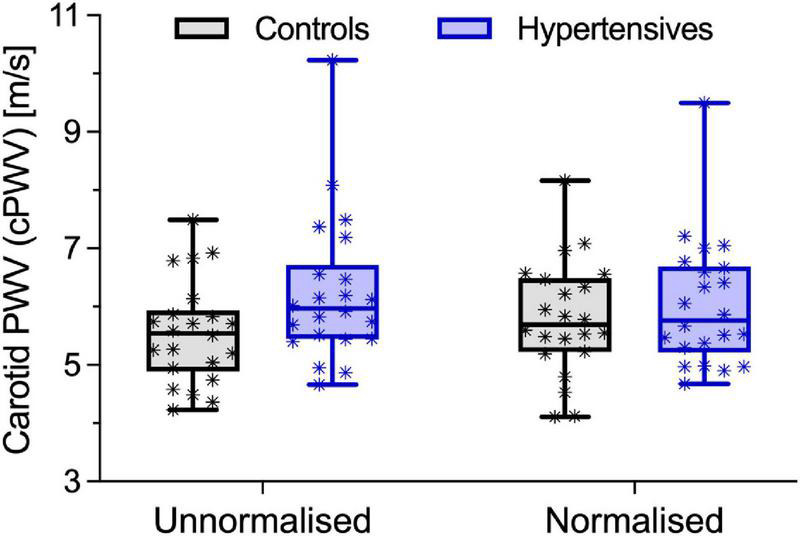
Local carotid pulse wave velocity (cPWV) in control and hypertensive people before and after pressure-normalization using Eq. 5. Bars indicate minimum and maximum.

**FIGURE 3:**
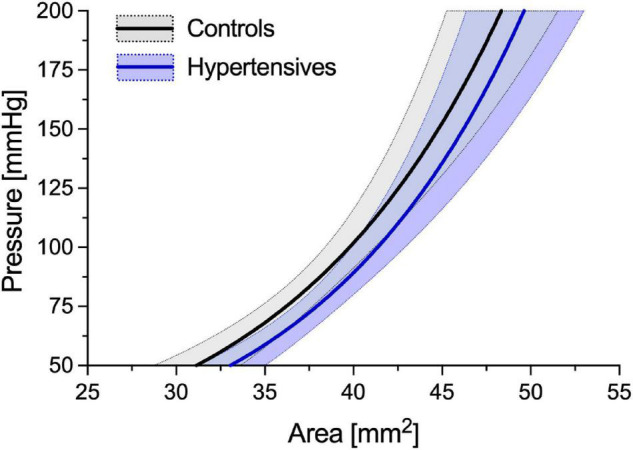
Average estimated *P*-*A* relationships of the control and hypertensive groups. Average curves have been built by estimating γ_0_ for all the subjects included in the two groups. Then, the subject-specific estimated *P*-*A* relationship was built using Eq. 1 and assuming a circular luminal area (i.e., *A* = π*D*^2^/4, where *D* is the diameter). Finally, ensemble averaging was performed between all people in each group. Solid lines indicate the average relationship, and areas delimited by dotted lines indicate ± standard deviation (SD).

As expected, before accounting for BP differences, cPWV, calculated using Eq. 6, was on average 12% higher in hypertensives than controls (6.24 ± 1.22 vs. 5.56 ± 0.86 m/s, *p* < 0.05) (Figure 2, left, and Table 2), even after HR adjustments (*p* < 0.05). However, the pressure-normalized stiffness index γ_0_ did not differ significantly (3.48 ± 1.04 vs. 3.77 ± 1.27, *p* = 0.41), suggesting that increased cPWV in hypertensives was likely mainly related to BP differences between groups. In fact, the average estimated *P-A* relationships of the two groups (Figure 3) run almost parallel to each other and are largely superimposed, suggesting that the mechanical response of the carotid arteries in the two groups was similar.

**TABLE 2:** Comparison between mechanistic and statistical blood pressure adjustment of the local carotid pulse wave velocity (cPWV) using different normalizing pressures.

	Controls		Hypertensives	
	Mechanistic	Statistical	Mechanistic	Statistical
Uncorrected	5.56 ± 0.86		6.24 ± 1.22	
SBP	5.88 ± 0.84	5.85 (5.40–6.30)	5.95 ± 0.98	5.95 (5.50–6.40)
MBP	5.82 ± 0.89	5.80 (5.34–6.26)	6.00 ± 1.00	6.00 (5.54–6.46)
*P*_*c*_	5.80 ± 0.95	5.77 (5.33–6.20)	6.04 ± 1.07	6.03 (5.54–6.46)
DBP	5.80 ± 0.94	5.69 (5.21–6.17)	6.03 ± 1.05	6.11 (5.59–6.47)

*DBP, diastolic blood pressure; MBP, mean blood pressure; P_c_, numerically determined local carotid PWV relevant pressure level; SBP, systolic blood pressure. Uncorrected and mechanistically corrected cPWV data are presented as mean ± standard deviation (SD). Statistically corrected cPWV data are presented as mean (95% confidence interval).*

### Pressure-Normalized Carotid PWV

*P*_*c*_, estimated from cPWV and γ_0_ by numerically solving Eq. 3, was 87.5 ± 10.3 in hypertensives and 78.4 ± 10.3 in controls, corresponding approximately to DBP + 0.085 (SBP–DBP) for both groups (Figure 3). Notably, in both groups, *P*_notch_ (Table 1) was, on average, (0.65 ± 0.10) PP above DBP. In a first BP-normalization, *P*_T_ was set to the average *P*_*c*_ across groups: 83.1 mmHg. After the BP-normalization, the difference in cPWV between the two groups reduced by 68%: 5.80 ± 0.94 m/s in controls and 6.03 ± 1.05 m/s in hypertensives (*p* = 0.47) (Figure 2, right). In fact, except for a single hypertensive subject whose cPWV largely exceeded those of controls both before and after the pressure-normalization, all other hypertensives had normalized cPWV within the control range. Similar results were obtained when normalizing using ANCOVA with *P*_*c*_ as a confounder: 5.77 (5.33–6.20) vs. 6.03 (5.59–6.47) m/s (*p* = 0.41) (62% of the total cPWV difference).

Table 2 reports the comparison between the BP-normalization using Eq. 5 and ANCOVA and using *P*_*c*_, SBP, MBP, and DBP as normalizing pressures. As expected, the corrections of cPWV were stronger when using higher values of normalizing pressure and, except when the normalizing pressure was set to DBP, showed good agreement between statistical and mechanistic methods. BP accounted for 90 (mechanistic) and 84% (statistical) with SBP as confounder, 75 and 69% with MBP, and 65 and 39% with DBP.

## Discussion

The BP-dependency of arterial stiffness limits the ability of PWV to define changes in the mechanical properties of the arterial wall in response to cardiovascular pathologies and wall remodeling and damage (Spronck, 2021). This fact assumes particular relevance when evaluating changes in arterial mechanics in response to hypertension since elevated BP drives the short-term increase in PWV and potentially the long-term damage to the wall microstructure that, in turn, could result in a chronic increase in PWV. The BP-dependency of PWV has been typically addressed *via* statistical methods (Desamericq et al., 2015; Valbusa et al., 2019). More recently, methods that rely on the exponential modeling of the wall behavior have allowed to mathematically predict subject-specific changes in PWV in response to acute changes in BP (Shirai et al., 2006; Spronck et al., 2017a). Using a similar approach, we aimed to characterize differences in CCA stiffness between healthy controls and hypertensive patients, providing subject-specific BP-normalization of cPWV. Furthermore, we aimed to compare the mechanistic BP-normalization with that obtained using statistical methods, as well as investigate the impact of the choice of the normalizing pressure on the obtained correction. Our results indicated that BP alone accounted for 68% of the cPWV difference between groups.

The association between hypertension and increased PWV has long been known (The Reference Values for Arterial Stiffness’ Collaboration, 2010). However, understanding the causal relationships between the two is less trivial; on the one hand, studies have shown that PWV is an independent predictor of the longitudinal increase in SBP (Najjar et al., 2008), so that increased PWV drives the development of hypertension. On the other hand, PWV intrinsically depends on the BP level at the time of the data acquisition, and the subject-specific PWV can vary considerably in response to BP changes (Spronck et al., 2015b). This two-way relationship between PWV and BP complicates investigating the consequences of increased BP on arterial mechanics. Methods for the BP-normalization of PWV aim to address this issue, providing stiffness metrics that refer to a predefined reference pressure level and are, hence, independent from the BP level at the time of the measurement (Spronck et al., 2017a; Giudici et al., 2021a).

In this study, we analyzed differences in CCA PWV between normotensive and Grade 1–2 hypertensive individuals. In agreement with previous studies (Maritz et al., 2016; Park et al., 2020), our results indicated that the cPWV was, in fact, higher in hypertensives than in controls at their relative working pressure: 12% difference with a 13 and 12% difference in SBP and DBP, respectively. Notably, while age and sex are key determinants of arterial stiffness (The Reference Values for Arterial Stiffness’ Collaboration, 2010), our studied groups were well-matched, so that these factors unlikely played a role in inter-group differences in cPWV. Our finding is in agreement with previous work (Laurent et al., 1994) where distensibility of the CCA at MBP was reduced by ∼33% in hypertensive people compared to healthy controls but with a ∼30% difference in SBP/DBP. Similar results have also been reported for regional carotid-femoral PWV (The Reference Values for Arterial Stiffness’ Collaboration, 2010). However, the pressure-normalization of cPWV proposed here indicated that more than two-thirds of this difference had to be imputed to BP differences between the two groups (Figure 2) and that, in absolute terms, the CCA of hypertensive patients was not intrinsically stiffer than that of healthy people. Notably, as suggested by the Moens-Kortweg equation (Moens, 1878; Li et al., 2021), PWV quantifies the structural stiffness of an artery as a whole (i.e., including both wall material stiffness and geometrical features). As hypertensives here had a higher IMT and IMT/*D*_d_ ratio than controls, residual differences in PWV after pressure-normalization may, at least in part, be attributable to these structural differences, rather than pure differences in wall material properties. This finding was further confirmed by the exponential modeling of the *P*-*A* relationships, with the average hypertensive curve running almost parallel to that of controls, independently of pressure. Our findings are in agreement with seminal works of Laurent et al. (1994) and Armentano et al. (1995), who showed that CCA distensibility-pressure relationships of controls and hypertensives are almost superimposed. These results suggest that wall stiffening in response to increased BP is mild at the CCA. It is worth considering, however, that patients in our study presented only mild-to-moderate hypertension with a relatively short time between the diagnosis of the condition and the time of the examination (< 1 year). Therefore, it is possible that longer exposure to severely increased BP might trigger intensive remodeling and, consequently, intrinsic stiffening (i.e., BP-independent) of the carotid wall.

**FIGURE 4:**
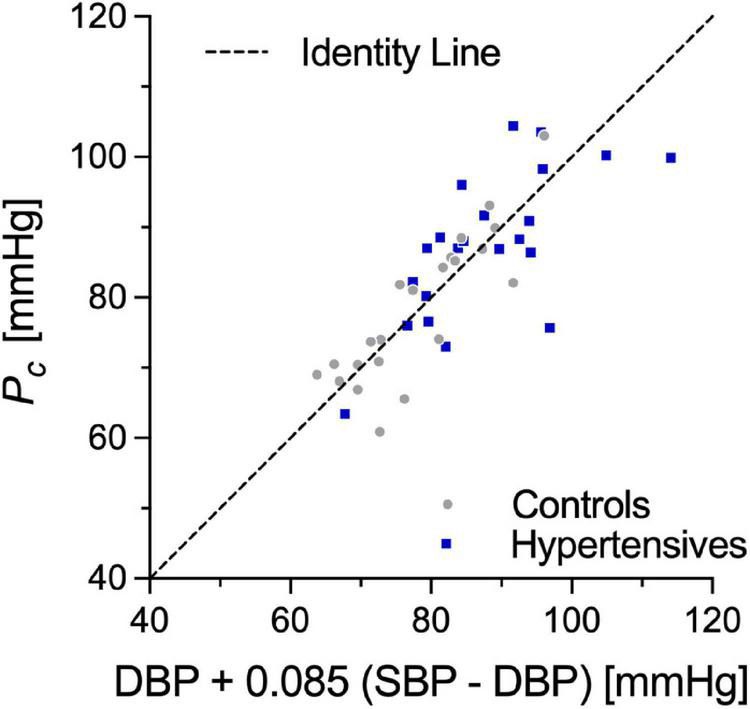
Correlation between *P*_*c*_, the pressure value linking cPWV to γ_0_, and DBP + 0.085 (SBP-DBP). SBP, systolic blood pressure; DBP, diastolic blood pressure.

Whether accounting for the BP-dependency of PWV with statistical or mechanistic methods, the choice of the normalizing pressure has an important quantitative impact on the size of the correction. While the most widely adopted choices in clinical studies are SBP or MBP (Desamericq et al., 2015; Valbusa et al., 2019), it has been previously argued that the BP-dependency of most metrics pertaining to PWV, especially those relying on the foot-to-foot detection technique, is likely governed by DBP (i.e., the pressure at the foot of the wave) rather than either SBP or MBP (Spronck et al., 2015b,2017a; Giudici et al., 2021a). In this study, cPWV was estimated *via* linear regression of the *P*-*D*^2^ relationship in late diastole, i.e., the diastolic decay after the dicrotic notch (Giudici et al., 2021c). Although this phase of the cardiac cycle spanned between DBP and ∼65% of the PP, our results indicated that, on average, *P*_*c*_ was determined for its 91.5% by DBP and only for its 8.5% by SBP (Figure 4). We obtained similar results when estimating cPWV *via* the ln*DU*-loop method, a linear regression between blood velocity (*U*) and the natural logarithm of the diameter distension (ln*D*) in early systole (Giudici et al., 2021d). These findings suggest that, as regional PWV, loop methods provide nearly diastolic measures of PWV and that DBP should be regarded as the main driver of their BP-dependency. Notably, however, in this study, DBP-adjustment provided the largest difference between statistical and mechanistic methods for the cPWV correction. This result warrants caution when performing statistical DBP-adjustment of all arterial stiffness metrics that are notably *purely* diastolic.

As other local techniques (Rabben et al., 2004; Khir and Parker, 2005; Feng and Khir, 2010; Campos Arias et al., 2019), the *D*^2^*P*-loop method (Alastruey, 2011) is based on the coupling of two arterial waveforms to provide an estimate of the wave speed at a single location of the arterial tree (i.e., local wave speed). In contrast, regional PWVs, such as the carotid-femoral PWV (Laurent et al., 2006), provide insights into the average arterial stiffness along a given wave path, which inevitably includes arteries with different wall structures. This is particularly relevant when considering that elastic and muscular arteries have shown to be affected differently by aging and disease (Borlotti et al., 2012). Furthermore, local methods are not affected by known limitations of regional PWVs, such as the possible inaccuracies in the estimation of the true arterial pathway length (Huybrechts et al., 2011; Giudici et al., 2021a). Finally, local arterial properties can be useful to predict damage of target organs located in the vicinity of the measurement site; for example, carotid stiffening and function have been associated with cognitive decline (Chiesa et al., 2019). For the aforementioned reasons, results reported here on differences in arterial wall stiffness between normotensives and hypertensives concern the carotid artery and should not be generalized to other locations or regions of the arterial tree.

## Limitations

In this study, we assumed the existence of an exponential *P*-*A* relationship for the CCA. While it is generally accepted that the *P*-*A* relationship of arteries closely resembles an exponential function in the physiological range of pressure (Gavish and Izzo, 2016), the subject-specific *P*-*A* relationships might not be exactly exponential, especially in young subjects at low pressures (Vande Geest et al., 2004). It is therefore possible that, for some subjects, the mechanistic method failed to accurately predict the relationship between BP and cPWV. It is, however, unlikely that this has significantly affected the cPWV-normalization since similar methods showed good ability in predicting changes in both local and regional PWV, given a BP change (Spronck et al., 2015b; Pucci et al., 2020).

## Conclusion

We concluded that mild hypertension does not chronically affect the stiffness of the CCA wall, at least in the middle-aged subjects without target organ damage.

Assuming an exponential *P*-*A* relationship, we proposed a method that allows for determining arterial stiffness, as PWV, independently of BP. The proposed method is non-invasive and provides a subject-specific normalization of PWV which could be implemented in the clinical setting. The results warrant further investigations to establish the potential clinical utility of the method.

## Data Availability Statement

The raw data supporting the conclusions of this article will be made available by the authors, upon reasonable request.

## Ethics Statement

The studies involving human participants were reviewed and approved by Comitato Etico di Area Vasta Nord Ovest (reference number: 3146/2010), Italy. The patients/participants provided their written informed consent to participate in this study.

## Author Contributions

AG contributed to the conceptualization, data analysis, manuscript drafting, and editing. AG and AK developed the analytical method. AK, CP, JC, and MK contributed to the conceptualization, manuscript editing, and project supervision. CM contributed to the data acquisition and management. All authors contributed to the article and approved the submitted version.

## Conflict of Interest

JC was a past president of the ARTERY (Association for Research into Arterial Structure and Physiology) society. MK was responsible for clinical studies at Esaote SpA (Genova, Italy). The remaining authors declare that the research was conducted in the absence of any commercial or financial relationships that could be construed as a potential conflict of interest.

## Publisher’s Note

All claims expressed in this article are solely those of the authors and do not necessarily represent those of their affiliated organizations, or those of the publisher, the editors and the reviewers. Any product that may be evaluated in this article, or claim that may be made by its manufacturer, is not guaranteed or endorsed by the publisher.

## APPENDIX

The tube law proposed (Eq. 1 in the main manuscript text) by Meinders and Hoeks (2004) relates changes in pressure (*P*) to those in the cross-sectional area (assumed circular: *A* = π*D*^2^/4):

(A1)
$$\begin{array}{c} P\,\left(D\right)=P_{\text{ref}}\,e^{\gamma_{0}\,\left(\frac{D^{2}}{D_{\text{ref}}^{2}}-1\right)}, \end{array}$$

where *P*_ref_ is a reference pressure, *D*_ref_ is the arterial diameter at *P*_ref_, and γ_0_ is an arterial stiffness index.

The Bramwell-Hill equation (Bramwell et al., 1923) indicates that pulse wave velocity (PWV) at a given pressure level *P*_*c*_ can be expressed as a function of the slope of the tangent to the *P*-*D*^2^ relationship at *P*_*c*_.

(A2)
$$\begin{array}{c} \text{PWV}\,\left(P_{c}\right)=\sqrt{{\frac{D^{2}}{\rho }\,\frac{\text{d}\,P}{\text{d}\,D^{2}}|}_{P_{c}}}, \end{array}$$

where ρ=1,060 kg/m^3^ is the blood density.

Given Eq. A1, the derivative term in Eq. A2 can be rewritten as follows:

(A3)
$$\begin{array}{c} {\frac{\text{d}\,P}{\text{d}\,D^{2}}|}_{P_{c}}=\frac{\text{d}}{\text{d}\,D^{2}}\,\left[P_{\text{ref}}\,e^{\gamma_{0}\,\left(\frac{D^{2}}{D_{\text{ref}}^{2}}-1\right)}\right]=\frac{P_{\text{ref}}\,\gamma_{0}}{D_{\text{ref}}^{2}}\,e^{\gamma_{0}\,\left(\frac{D_{c}^{2}}{D_{\text{ref}}^{2}}-1\right)}, \end{array}$$

where *D*_*c*_ is the diameter value corresponding to the pressure level *P*_*c*_ [i.e., *P*_*c*_ = *P*(*D*_*c*_)]. Inverting Eq. A1 to express diameter as a function of pressure leads to the following relationship:

(A4)
$$\begin{array}{c} D=D_{\text{ref}}\,\sqrt{1+\text{ln}\,\frac{\left(\frac{P}{P_{\text{ref}}}\right)}{\gamma_{0}}}, \end{array}$$

so that *D*_*c*_ becomes as follows:

(A5)
$$\begin{array}{c} D_{c}=D_{\text{ref}}\,\sqrt{1+\frac{\text{ln}\,\left(\frac{P_{c}}{P_{\text{ref}}}\right)}{\gamma_{0}}}. \end{array}$$

Substituting Eq. A5 in Eq. A3 yields as follows:

(A6)
$$\begin{array}{c} {\frac{\text{d}\,P}{\text{d}\,D^{2}}|}_{P_{c}}=\frac{P_{\text{ref}}\,\gamma_{0}}{D_{\text{ref}}^{2}}\,e^{\gamma_{0}\,\left(\frac{\text{ln}\,\left(\frac{P_{c}}{P_{\text{ref}}}\right)}{\gamma_{0}}\right)}=\frac{P_{c}\,\gamma_{0}}{D_{\text{ref}}^{2}}. \end{array}$$

Replacing the derivative in Eq. A1 with Eq. A6 and rearranging using Eq. A5 then leads to Eq. A7 (corresponding to Eq. 3 in the main manuscript text):

(A7)
$$\begin{array}{c} \text{PWV}\,{\left(P_{c}\right)}^{2}=\frac{D_{c}^{2}}{\rho }\,\frac{P_{c}\,\gamma_{0}}{D_{\text{ref}}^{2}}=\left(1+\frac{\text{ln}\,\left(\frac{P_{c}}{P_{\text{ref}}}\right)}{\gamma_{0}}\right)\,\frac{P_{c}\,\gamma_{0}}{\rho }=\frac{P_{c}\,\gamma_{0}}{\rho }+\frac{P_{c}}{\rho }\,\text{ln}\;\left(\frac{P_{c}}{P_{\text{ref}}}\right). \end{array}$$
